# Opioid Facilitation of β-Adrenergic Blockade: A New Pharmacological Condition?

**DOI:** 10.3390/ph8040664

**Published:** 2015-09-25

**Authors:** Joseph Vamecq, Karine Mention-Mulliez, Francis Leclerc, Dries Dobbelaere

**Affiliations:** 1 Inserm, Laboratory of Molecular Biology and Biochemistry, Hormonology Metabolism Nutrition and Oncology, Center of Biology and Pathology, CHRU Lille, and RADEME EA 7364, Faculty of Medicine, University of Lille 2, Lille 59037, France; 2 Centre de Référence Maladies Héréditaires du Métabolisme de l’Enfant et de l’Adulte, Jeanne de Flandre Hospital, CHRU Lille, and RADEME EA 7364, Faculty of Medicine, University Lille 2, Lille 59037, France; E-Mails: karine.mention@chru-lille.fr (K.M.-M.); dries.dobbelaere@chru-lille.fr (D.D.); 3 Pediatric Critical Care Unit, Hôpital Jeanne de Flandre, CHRU Lille, Lille 59037, France; E-Mail: francis.leclerc@chru-lille.fr

**Keywords:** lactate, glycolysis disruption, Na^+^/K^+^ ATPase, β-adrenergic receptor, G protein, cAMP, protein kinase A, shock, δ-opioid receptor

## Abstract

Recently, propranolol was suggested to prevent hyperlactatemia in a child with hypovolemic shock through β-adrenergic blockade. Though it is a known inhibitor of glycolysis, propranolol, outside this observation, has never been reported to fully protect against lactate overproduction. On the other hand, literature evidence exists for a cross-talk between β-adrenergic receptors (protein targets of propranolol) and δ-opioid receptor. In this literature context, it is hypothesized here that anti-diarrheic racecadotril (a pro-drug of thiorphan, an inhibitor of enkephalinases), which, in the cited observation, was co-administered with propranolol, might have facilitated the β-blocker-driven inhibition of glycolysis and resulting lactate production. The opioid-facilitated β-adrenergic blockade would be essentially additivity or even synergism putatively existing between antagonism of β-adrenergic receptors and agonism of δ-opioid receptor in lowering cellular cAMP and dependent functions.

## 1. Introduction

In a previous report, the prevention of hyperlactatemia development in a nine-month-old child undergoing a shock directly caused by dehydration was put in relation with exposure to propranolol, a drug not usually indicated in shock, but here given as a chronic therapy to treat hemangioma [1]. Main clinical features of this patient under shock are summarized in Table 1. Anti-hyperlactatemia mechanisms proposed in this circumstance were propranolol-driven β-adrenergic blockade in skeletal muscle, a key organ in regulation of lactate overproduction [1].

**Table 1:** Main clinical features and therapeutic measures in a child undergoing a hypovolemic shock caused by dehydration ^a^.

Clinical Period	Clinical Signs
Before shock	The girl child has a Cyrano hemangioma and is treated since 4 months of age with propranolol (3 × 15 mg/day)
Onset and outcome of shock	At 9 months, she develops an acute episode of rotavirus gastroenteritis complicated by profuse diarrheas and drowsiness and treated with oral rehydration solution and racecadotril (10 mg/day). The child becomes shocked and dehydrated, presenting with mottled skin, cold extremities, capillary-refill time of 5 s, weight of 7.5 kg, heart rate at 100 beats/min, arterial pressure at 62/21 mmHg, and respiratory frequency of 40 cycles/min. Biology (normal range values between rounded brackets) indicates severe hypernatremia 163 mmol/L (137–145 mmol/L), hypokaliemia 2.2 mmol/L (3.5–4.5 mmol/L), elevated blood chloride 138 mmol/L (102–108 mmol/L), acidic blood pH at 6.80 (7.35–7.45) low CO_2_ partial pressure at 20 mmHg (35.7–44.8), a negative base excess at −25 mEq/L (−2 to +2 mEq/L) and hypocalcemia at 69 mg/L [total calcium 92–106 mg/L]. Blood protein level is normal at 70 g/L) as well as glycemia at1.31 g/L, uremia at 0.45 g/L or 7.5 mmol/L, creatininemia at 6 mg/L or 53 µmol/L and blood lactate at 0.8 mmol/L [0.66–2.4 mmol/L]. Hemoglobin at 9.5 g/100 mL [12.5–21.5 g/100 mL] indicates anemia. Fluid resuscitation initiated by intraosseous route followed by intravenous rehydration resolves hypovolemic shock while blood ionogram and pH partially improved, lactatemia staying normal (0.7 mmol/L). Intravenous rehydration is continued and bicarbonate administered. Dehydration signs, hypernatremia and acidosis gradually resolve, patient recovering without sequelae.

^a^ The patient was subject to a previous case report [1].

**Figure 1:**
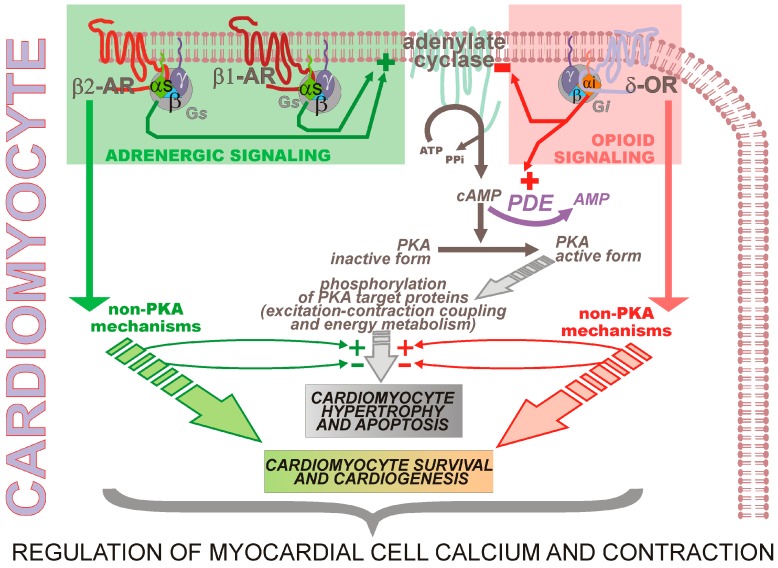
Cross-talk between β-adrenergic and δ-opioid signaling pathways in cardiomyocytes as an experimental basis for regulation of myocardial cell calcium and contraction.

Even though previously proposed mechanisms were coherent, a facilitation of the β-blocker effects is here considered for three main reasons:

- Though it is a known inhibitor of glycolysis, propranolol, outside this previous observation, has never been reported to fully protect against lactate overproduction.
- During shock, the patient was treated by the anti-diarrheic racecadotril [1], a pro-drug of the enkephalinase inhibitor thiorphan [2,3] acting via a rise in enkephalins and hence a stimulation of opioid receptor signaling, conferring a reduced intestinal secretory activity [4,5,6,7,8,9,10,11,12,13].
- Importantly, previous experimental support has been given in favor of a cross-talk between β-adrenergic receptors and δ-opioid receptor signaling [14,15,16] (Figure 1).

Experimental evidence for the depicted events has been previously given [15,16] and reviewed [14] through works from which the figure is also adapted. In brief, β-adrenergic and δ-opioid receptors modulate Protein kinase A (PKA) and non-PKA mechanisms involved in cell calcium and excitation-contraction coupling. Whereas, as a whole, β-adrenergic receptors positively impact adenylate cyclase and then stimulate PKA and resulting activation of PKA target proteins, δ-opioid receptor acts in an inverse way, blunting recruitment of PKA and target proteins. Not only adenylate cyclase is inhibited but also concomitant activation of phosphodiesterase activity (PDE) by δ-opioid receptor further strengthens the negative signaling operated on PKA activation through removal of cAMP in the form of AMP. In parallel with PKA-dependent pathways, non-PKA mechanisms are mediated by β-adrenergic and δ-opioid receptors in the control of myocardial cell calcium and contraction. These mechanisms may either amplify or reduce PKA-dependent signaling, in the same time as mediating other effects. More especially and not illustrated, the β2-adrenergic receptor, like the δ-opioid receptor, is coupled to a Gi protein and in both cases the βγ subunits of the Gi proteins may activate RAS/MAPK signaling and PI3K [14].

Abbreviations are: β1-AR, β1-adrenergic receptor; β2-AR, β2-adrenergic recptor; δ-OR, δ-opioid receptor; Gs, Gs-protein; Gi, Gi-protein; ATP, adenosine triphosphate; AMP, adenosine monophoshate; cAMP, cyclic AMP; PPi, inorganic pyrophosphate; PKA, proteine kinase A; PDE, phosphodiesterase; + and −, stimulation and inhibition, respectively.

Keeping in mind these aspects, opioid-facilitated β-adrenergic blockade, more especially facilitation of the metabolic effects of propranolol by racecadotril, is presented thereafter as a new pharmacological condition mainly based on the known cross-talk between β-adrenergic and δ-opioid signaling pathways, being aware of other cross-talks between adrenergic and opioid (α-adrenergic and δ-opioid [17], β-adrenergic and κ-opioid [18]) signaling pathways. The facilitation of β-adrenergic blockade by opioid signaling essentially lies in the fact that notably δ-opioid activation decreases levels of the second messenger (cAMP), the depression of which mediates the cellular effects of β-adrenergic blockade. Preliminary brief introductions are meanwhile given to fast glycolysis, β-adrenergic control of lactate production and effects of propranolol on glycolysis.

## 2. Fast Glycolysis and Lactate Production

Substantial lactate production by cells is the result of a glycolysis not relayed by mitochondrial oxidations. In these conditions, as a result of limited amounts of ATP produced per molecule of glucose, glycolysis proceeds at fast rates to yield sufficient ATP to cover cell energetic needs. Anaerobic glycolysis includes physiological glucose oxidations taking place when either mitochondrion are absent (erythrocytes) or when oxygen supply to mitochondrion is swamped (e.g., sustained and intense muscle contractions). Anaerobic glycolysis may also develop in pathophysiological hypoxic/anoxic conditions. When taking place in normoxic conditions, glycolysis may be relayed by mitochondrial oxidations and due to an overall improvement of ATP production per molecule of glucose, this glycolysis occurs at a slow pace with little or no lactate production. Nevertheless, even in such normoxic conditions, glycolysis may be not relayed by mitochondrial oxidations, and for this reason may also proceed at fast rates with substantial lactate production. This occurs when mitochondrial oxidations are deficient or when they are regulated negatively under biased signaling such as observed in the Warburg’s effect [19]. Because like anaerobic glycolysis, it exhibits intensive glucose utilization, this biased normoxic glycolysis is called aerobic glycolysis. [19].

## 3. Adrenergic Signaling and Lactate Production

Lactic acid, in the ionized lactate form at physiological pH, is a glycolysis byproduct that increases in blood under tissue hypoxia and other conditions reviewed elsewhere [20]. Lactate formation attests for the activity of the anaerobic glycolysis, and therefore occurs when anaerobic glycolysis physiologically (e.g., rapid and sustained muscle contraction) or pathophysiologically (e.g., tissue hypoperfusion) develops. Under tissue hypoperfusion, the reliability of lactate as a blood marker of shock severity, however, still remains debated; lactate production having been supported to depend on adrenergic response rather than on shock severity itself [21] and, in sepsis, to be the result of increased pyruvate formation rather than decreased oxygen availability [22]. The link between adrenergic cell signaling and blood lactate has been experimentally shown in an animal model of hemorrhagic shock [20], before being proposed in the humans [23]. In shock, catecholamines are increased, and taking into account that epinephrine potently stimulates lactate formation under well-oxygenated conditions, adrenergic signaling has been proposed to contribute, besides tissue hypoperfusion, to the rise of lactate which may be observed during shock [21,24,25]. In rat muscle, epinephrine and other agonists of β-adrenergic receptors stimulate glycolysis but also membrane polarization, cell sodium efflux and potassium influx, this decreased intracellular Na^+^ to K^+^ ratio attesting for stimulation of the Na^+^/K^+^ ATPase pump [26,27]. Interestingly, propranolol, which inhibits β-adrenergic receptors, and ouabain, which inhibits the Na^+^/K^+^ ATPase pump, are capable of blunting epinephrine-stimulated glycolysis [26,28,29,30]. On the basis of this experimental background, additional support has been given to link increased muscle glycolysis (the main producer of circulating lactate) to epinephrine-stimulated Na^+^/K^+^ ATPase pump activity [21]. The fact that β-adrenergic blockers such as propranolol may aggravate increased circulating glucose and insulin resistance in diabetic patients may be consistent with a link between β-adrenergic blockade and disrupted glycolysis [31]. Finally, the best support to link lactate formation and adrenergic signaling might perhaps lie in compelling evidence stressing the ability of the β-adrenergic agonist therapy to rise lactatemia in asthmatic and healthy subjects [32,33,34,35,36,37,38,39,40,41,42].

## 4. β-Adrenergic Blockade by Propranolol Decreases Lactate Production in a Non-Septic Shock

Though sometimes described in septic shock [43], the lack of hyperlactatemia during decompensated hypovolemic shock caused by dehydration represents, in contrast, a rather unique event [1]. In this respect, we have recently described a child developing shock caused by dehydration during the course of a viral gastroenteritis and without hyperlactatemia [1]. In this case, prevention of hyperlactatemia was attributed to adrenergic blockade by propranolol [1].

## 5. Facilitation of Propranolol-Driven β-Adrenergic Blockade by Racecadotril

Though proposed underlying mechanisms (reduction of cAMP and hence drop in both PKA-mediated phosphorylation of phospholemman and subsequent stimulation of Na^+^/K^+^ ATPase) [1] were coherent, prevention of lactate over production by propranolol still remains a unique observation. This led us to review all other factors that might have helped the drug to prevent hyperlactatemia in our previous observation. Though no link with glycolysis disruption was previously suggested in the literature for the anti-diarrheic racecadotril (a prodrug of thiorphan, an enkephalinase inhibitor [2,3]), a detailed analysis of its signaling effects suggests some putative cross-talk with signaling triggered by propranolol. In fact, δ-opioid receptor activation by enkephalins accumulating under racecadotril might facilitate β-blocker effects as regards to lactate production. The notion of “opioid-facilitated β-adrenergic blockade” is here proposed to underline the particular pharmacological context induced by the concurrent administration of the two drugs. The mechanisms by which this situation may negatively impact glycolysis rates are presented thereafter essentially through the abilities of the two drugs, racecadotril and propranolol, to act in concert in lowering cell cAMP levels.

## 6. Opioid-Facilitated β-Adrenergic Blockade as a Mean to Prevent Hyperlactatemia

**Figure 2:**
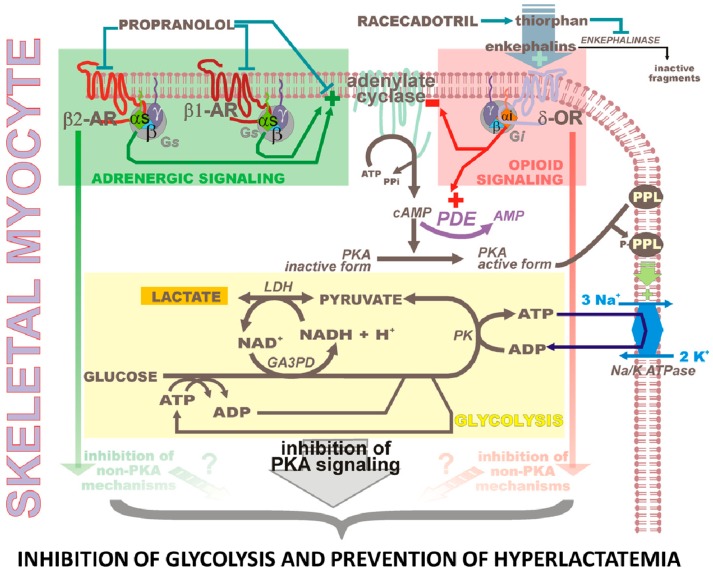
Cross-talk between β-adrenergic and δ-opioid signaling pathways in skeletal myocytes as a theoretical basis for reinforced inhibition of glycolysis and prevention of hyperlactatemia.

Previous support for propranolol-driven inhibition of lactate production [1] is explained thereafter. β-adrenergic blockade may be achieved by propranolol, a non-selective blocker of β1 and β2 adrenergic receptors. Though β2-adrenergic receptor may also activate Gi proteins-driven signaling, these β-adrenergic receptors are coupled to G*s* proteins that activate adenylate cyclase [44]. Subsequent rises in intracellular cAMP level and protein kinase A activity (PKA) induce enhanced phosphorylation of phospholemman (FXYD1, a member of FXYD protein family) and stimulation of plasma membrane Na^+^/K^+^ ATPase in organs including heart, liver and skeletal muscle [45]. This ATPase by hydrolyzing ATP to ADP maintains a high cytosolic ADP/ATP ratio stimulating glycolysis, pyruvate and hence lactate production. By blocking β-adrenergic receptors, propranolol prevents cytosolic rise in cAMP and activation of PKA induced by catecholamines. Phospholemman phosphorylation and its stimulatory effect on Na^+^/K^+^ ATPase activity are consequently prevented, reducing ATP hydrolysis, ADP/ATP ratio and glycolytic rate (Figure 2), a scenario in agreement with experimental inhibition by adrenergic blockade of lactate production [23]. As mentioned above, racecadotril (acetorphan) is a pro-drug of thiorphan. This enkephalinase inhibitor increases steady-state concentrations of enkephalins in gut and signaling via δ-opioid receptor, lowering intracellular cAMP and hence ion and water secretory activity of enterocytes [4,5,6,7,8,9,10,11,12,13]. These changes reverse enterocyte high cAMP levels causative of diarrhea [11]. Racecadotril produces significant inhibition of plasma enkephalinases after oral administration [7], likely stimulating opioid signaling in δ-opioid receptors-containing organs. These organs involving heart also include skeletal muscle [46,47], which therefore also represents a *bona fide* target for endogenous enkephalins accumulating secondarily to inhibition of enkephalinases. Resulting drop in intracellular cAMP would mimic β-adrenergic blockade making δ-opioid receptor stimulation a potentiator of adrenergic blockade in skeletal muscle (Figure 2).

This particular pharmacological context is attributed to the combined administration of propranolol and racecadotril in a patient in severe hypovolemic shock caused by dehydration [1]. For abbreviations, see Figure 1 or below in this legend. β1- and β2-adrenergic receptors (β1-AR and β2-AR, respectively) are coupled to Gs proteins (“s” for stimulation of adenylate cyclase) and δ-opioid receptor (δ-OR) to a Gi protein (“i” for inhibition of adenylate cyclase). Propranolol β-adrenergic blockade prevents the increase of plasma membrane adenylate cyclase activity and intracellular cAMP levels induced by catecholamines. Racecadotril induces the inhibition of adenylate cyclase via activation of δ-OR. The resulting cumulated drop in cAMP prevents the activation of protein kinase A (PKA) and phosphorylation of phospholemman (PPL), alleviating the stimulatory effect of this membrane protein on the activity of Na^+^/K^+^ ATPase. Decreasing the contribution of ATPase to recycle cytosolic ADP from ATP explains why cytosolic ADP/ATP ratio lowers and why glycolysis rate is slowed by the drugs. One glucose gives rise to 2 pyruvate, and the 2 ATP consumed during the first steps of glycolysis (glucokinase/hexokinase(s) and phosphofructose kinase 1) are recovered at the end of the phosphoglycerate kinase-catalyzed reaction. Only the ATP molecules generated by pyruvate kinase (PK) are considered to provide cells with a net gain in ATP formation during glycolysis. As illustrated by the figure, this is this pool of ATP that needs to be converted back to ADP in order to allow continuation of glycolysis. Note that NAD^+^ (cofactor oxidized form) needs also to be recycled (from NADH + H^+^ (cofactor reduced form)) to maintain glycolysis. In the figure, the recycling is illustrated to be ensured essentially by lactate dehydrogenase (LDH). This occurs when alternative ways to consume cytosolic NADH are substantially switched off, *i.e.*, when mitochondrial oxidation of pyruvate is deficient, notably under hypoxia/anoxia (by lack of oxygen), inflammatory and truncated HIF signaling conditions. Shock is a condition that may combine inflammatory and hypoxic conditions, and then which favors increased cellular production of lactate and hyperlactatemia. Auxiliary systems that need to be recruited to optimize the underlying stimulation of glycolysis include those that maintain the cytosolic ADP/ATP ratio at high levels. In this exercise, exacerbated membrane Na^+^/K^+^ ATPase activity is essential. In fact, propranolol and racecadotril acting synergistically to alleviate recruitment of the Na^+^/K^+^ ATPase activity defeat the whole sketch, which in shock leads to exacerbated glycolysis, lactate production and hyperlactatemia.

## 7. Additivity or Synergism of Drug Actions?

A last but not least aspect of the proposed new concept holds in the nature of how each of the drug actions on glycolysis may act in concert. Additivity refers to drug mechanisms just adding, so as a net result, the intensity of the action of the combined drugs corresponds to the expected sum of each individual drug activity. Synergism refers to a net result which differs from that expected from simply the sum of each of the individual drug activity. Analysis of data on the cross-talks between β-adrenergic and δ-opioid receptors might suggest synergism in heart since the effects of local stimulation of β-adrenergic receptor are attenuated by positive opioid signaling at ineffective concentrations [48]. In the scope of impacting skeletal muscle metabolism involved in the control of body lactate production, one might suggest that some synergism also takes place in the pharmacological concept illustrated in Figure 2. Indeed, in addition to the sum of the expected decreases in productions of cAMP induced by blockade of β-adrenergic receptors and activation of δ-opioid receptor through the drop in adenylate cyclase activity, the δ-opioid receptor may further influence PKA activation by a stimulation of phosphodiesterase action and resulting enhancement in cAMP degradation, then potentiating the direct decrease in cAMP production. Modulation of PKA effects through the signaling triggered in parallel by δ-opioid receptor activation in non-PKA dependent mechanisms (Figure 1 and Figure 2) is not ruled out. These non-PKA mechanisms have been mentioned above in the case of the cross-talk between β-adrenergic and δ-opioid signaling pathways taking place in the heart. Regarding other cross-talks also mentioned above for heart function, between α-adrenergic and δ-opioid [17] and between β-adrenergic and κ-opioid [18] signaling pathways, they might be, on the opposite, *a priori* considered of a limited importance in our proposed concept because propranolol is a β-adrenergic blocker [49,50] and skeletal muscle does not exhibit κ-opioid receptors [51], respectively.
